# A pan-cancer agent for screening, resection and wound monitoring via NIR and SWIR imaging

**DOI:** 10.21203/rs.3.rs-3879635/v1

**Published:** 2024-01-23

**Authors:** Benedict Mc Larney, Ali Sonay, Elana Apfelbaum, Nermin Mostafa, Sébastien Monette, Dana Goerzen, Nicole Aguirre, Elizabeth Isaac, Ngan Phung, Magdalena Skubal, Mijin Kim, Anuja Ogirala, Darren Veach, Daniel Heller, Jan Grimm

**Affiliations:** Memorial Sloan Kettering Cancer Center; Memorial Sloan Kettering Cancer Center; Memorial Sloan Kettering Cancer Center; Memorial Sloan Kettering Cancer Center; Memorial Sloan Kettering Cancer Cen; Memorial Sloan Kettering Cancer Center; Memorial Sloan Kettering Cancer Center; Memorial Sloan Kettering Cancer Center; Memorial Sloan Kettering Cancer Center; Memorial Sloan Kettering Cancer Center; Memorial Sloan Kettering Cancer Center; Memorial Sloan Kettering Cancer Center; Memorial Sloan Kettering Cancer Center; Memorial Sloan Kettering Cancer Center; Memorial Sloan Kettering Cancer Center

## Abstract

Fluorescence guided surgery (FGS) facilitates real time tumor delineation and is being rapidly established clinically. FGS efficacy is tied to the utilized dye and provided tumor contrast over healthy tissue. Apoptosis, a cancer hallmark, is a desirable target for tumor delineation. Here, we preclinically in vitro and in vivo, validate an apoptosis sensitive commercial carbocyanine dye (CJ215), with absorption and emission spectra suitable for near infrared (NIR, 650–900nm) and shortwave infrared (SWIR, 900–1700nm) fluorescence imaging (NIRFI, SWIRFI). High contrast SWIRFI for solid tumor delineation is demonstrated in multiple murine and human models including breast, prostate, colon, fibrosarcoma and intraperitoneal colorectal metastasis. Organ necropsy and imaging highlighted renal clearance of CJ215. SWIRFI and CJ215 delineated all tumors under ambient lighting with a tumor-to-muscle ratio up to 100 and tumor-to-liver ratio up to 18, from 24 to 168 h post intravenous injection with minimal uptake in healthy organs. Additionally, SWIRFI and CJ215 achieved non-contact quantifiable wound monitoring through commercial bandages. CJ215 provides tumor screening, guided resection, and wound healing assessment compatible with existing and emerging clinical solutions.

## Introduction

Surgery is often the first and sometimes (ideally) the only treatment a cancer patient will undergo. Tumor surgery is evolving but relies on a surgeon’s ability to innately determine tumor from healthy tissue under visible light inspection. Fluorescence guided surgery (FGS) improves tumor identification and delineation by visualizing fluorescent dyes selectively accumulating in tumors. Fluorescence imaging and FGS are heavily used preclinically but not yet widely adopted clinically, with multiple barriers to be overcome by compounds and devices.^1^ These limitations include the lack of required expertise in clinical domains and ultimately the need to prove utility to patient outcomes, despite the clinical dominance of optical techniques.^2^ Targeted and passive fluorophore accumulation imaged via FGS can dramatically improve a surgeon’s capability to resect a tumor, aid in finding smaller lesions, identify lesions not detected by conventional methods, and locate lesions which move between pre-op scans and surgery (e.g. bowel-associated).^3,4^ With the advent of various FGS-based systems, especially those within robotic guided surgery, dyes which can readily be adapted to current clinical workflows are of the utmost importance.^1^ Atumor selective antibody conjugated to a fluorophore of choice (e.g., indocyanine green (ICG)) is the predominant approach for FGS, providing robust results, but is often only specific for a single-tumor type.^5–9^ Therefore, a novel compound must be developed for each tumor type to be targeted, preclinically tested and then subject to clinical approval, in a time (over a decade) and financial (~$100–200 million) very costly process.^10,11^ The “by the minute” charges for surgical room use (avg. USD$46.04/min in 2022) where FGS requires additional time further hinders translation, preventing preclinically validated fluorophores from having the highest possible impact.^3,12,13^ FGS patients normally receive a single dose of the compound, and this (combined with the high costs of bringing such a compound to market) provides yet another barrier to translation.^10,14^ To overcome this pan-tumor agents are the of some of the highest value in biomedical cancer research, not only for FGS, utilizing e.g., the known lower pH environment of most tumors.^15^

Another long-established hallmark of cancer is apoptosis where unregulated cell reproduction and growth results in cellular damage and death.^16–18^ Cellular damage and apoptosis are highest within the core of a tumor, where hypoxia and reduced blood flow form a non-sustainable environment.^19,20^ Efforts to clinically leverage this feature for FGS are overdue, with few promising preclinical results so far.^21–24^ Current solutions have neither been clinically translated nor are they produced in larger quantities. There is a clear demand for a scalable solution which should be easy to use, handle, and administer. Additionally, a dye sensitive to apoptosis could potentially have minimal uptake and therefore background in healthy tissue, improving tumor delineation via clearance from non-cancerous (non-damaged) areas. This would inherently provide a pan-cancer targeting approach for numerous clinical applications, and potentially scenarios outside of tumor resection where apoptosis plays a role. Having multiple dye applications could offset current market barriers and improve patient outcomes.^25^

In this work, we have investigated the potential for delineation of multiple solid tumors and monitoring of wound healing *in vivo* via a single NIR/SWIR-emitting small molecule dye, CJ215. CJ215 is a commercial, carbocyanine dye, with a molecular weight of ~1330g/mol and is inherently tumor targeting without antibody, peptide or ligand conjugation.^26^ CJ215 has a strong NIR fluorescence band (absorbance from 795–820 and peak emission from 810–840 depending on solution), and like ICG, extends to the SWIR region, potentially useful for SWIRFI in clinical settings. We show cellular localization of CJ215 and its general *in vivo* tumor specificity. CJ215 was taken up and delineated multiple preclinical solid tumor lines including breast (4T1, murine derived), prostate (PC3-PSMA, human derived), colon (CT26, murine derived), fibrosarcoma (HT1080, human derived) and finally, intraperitoneal colorectal cancer (SW1222, human derived).^27–30^ These models represent some of the most prevalent and fatal tumors, where surgery will be a primary a treatment option (with or without adjuvant chemo/radiation therapy).^31–35^ CJ215 tumor uptake could be detected with both SWIRFI and NIRFI. SNR and CNR assessment with SWIRFI were performed during screening and resection at >900 nm (sensor response) for all tumor lines and additionally at >1100 and >1300 nm long pass cut offs (via long pass filters) for two tumor lines. Finally, apoptosis is also a known component of wound healing. We therefore evaluated CJ215 for quantitative non-contact and ambient light resistant longitudinal wound assessment through commercial bandages without removing them. CJ215 wound uptake and clearance was in agreement with reported healing timelines, with a repeat injection post healing not delineating the then healed wound.^36–38^ This work establishes CJ215 not only as a pan cancer agent, which can delineate multiple solid tumors with a single systemic intravenous injection but also an agent with applications beyond tumor surgery. We further take advantage of the transparency of plastic compounds in the SWIR region to enable SWIRFI for wound assessment through bandages.^39^ This work aids the establishment and translation of fluorescence for clinical domains, extending beyond FGS and into post-operative care.

## Results

### Spectral characterization of CJ215:

**Figure 1:**
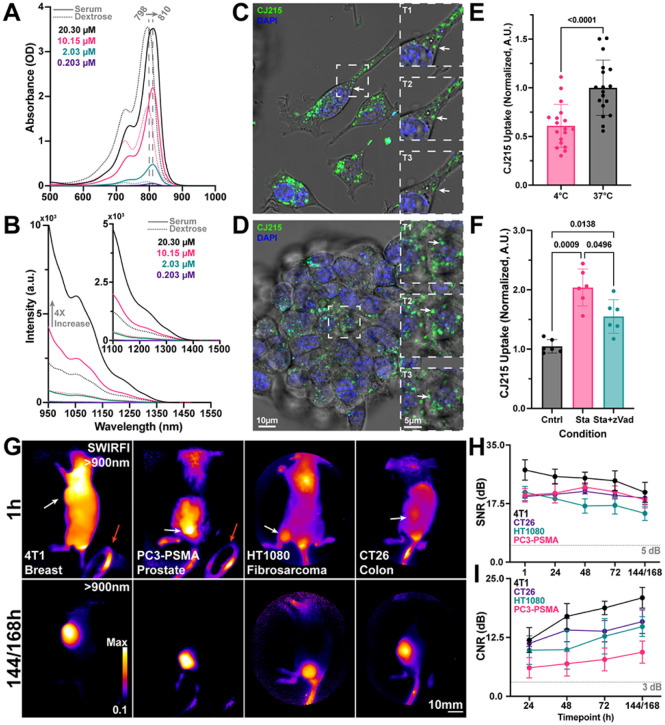
CJ215 spectral, in vitro, and in vivo assessment. **A)** The absorption of CJ215 dissolved in either fetal bovine serum (solid line) or dextrose (dotted line) was assessed at concentrations from 20.30 μM to 0.203 μM. The entire absorption spectrum red shifted in serum 12nm from a peak of 798 to 810 nm, like that of ICG. **B)** The SWIR emission tail of the dye was characterized from 950 to 1550 nm with notable emission past 1100 nm and a 4x increase in intensity when dissolved in serum vs dextrose. **C)** Representative single cell NIR confocal microscopy localization uptake of CJ215 (green, 3hr incubation period) in 4T1 cells, stained with DAPI (nucleus) with polarized white light images overlayed (grayscale). Inset, zoom in of vesicles containing CJ215 and their transport within the cell highlighting vesicle localization (T1=0s, T2=+23.175s, T3=+38.625s). **D)** Representative Z-stack slice of CJ215 uptake in 4T1 spheroids (green, 3hr incubation period), inset, zoomed in image highlighting vesicle transport over time (T1=0s, T2=+17.962s, T3=+35.924s). Supplemental videos 1 and 2 further show vesicle transport. **E)** CJ215 uptake in 4T1 cells is based upon active transport with cells incubated with the dye at 37°C showing significantly increased (39.067%, Welch’s two sided, unpaired, parametric t-test) uptake compared to those incubated with CJ215 at 4°C, mean and standard deviation (SD) are shown based on n=18 technical replicates from n=3 biological replicates (6 well plates). **F)** Cell damage and apoptosis induction by staurosporine (Sta) significantly increases CJ215 uptake in HT1080 cells by 99.298% compared to control cells. zVad-FMK (zVAD, an apoptosis blocker) combined with Sta reduced maximum uptake (48.826%) compared to Sta alone. Mean, SD, and replicates (dots) are shown with statistical analysis shown based on a non-paired or matched Welch and Brown-Forsythe ANOVA with Dunnet T3 multiple comparisons test. **G)** Ambient light resistant SWIRFI based (>900 nm) screening of four tumor cell lines including 4T1 (breast, orthotopic, murine), PC3-PSMA (prostate, heterotopic, human), HT1080 (fibrosarcoma, orthotopic, human) and CT26 (colon, heterotopic, murine). Top, representative images of all tumor lines 1 hr post intravenous systemic injection of CJ215. Fluorescence reflections are visible in the ambient lighting LED (red arrow, circular object in bottom right). Bottom, images of tumor uptake and localization of CJ215 144 or 168 hrs post injection at video rate exposures (1–10ms). In all cases the tumor can be readily delineated. **H)** Signal to noise ratio (SNR, dB) quantification of all tumor lines. At all timepoints sufficient SNR is achieved above the 5dB threshold with exposure times ranging from 1–2 ms (808nm excitation, ~300 mW/cm^2^). **I)** Contrast to noise ratio (CNR, dB) quantification for all tumors and all mice, where sufficient contrast is achieved (above 3dB threshold) 24hrs post injection with CNR steadily increasing up to 144/168 hrs for all tested tumor lines. In all cases the mean (dot) and SD are shown from n=4 mice per tumor line (n=16 mice total).

CJ215 is a small molecule carbocyanine dye with a molecular weight of approx. 1330g, **Supp. 1**. First, we pro led the spectral absorption of CJ125 and determined whether it had any SWIR emissive tail, as has been demonstrated for ICG and ICG conjugated cancer targeting probes, Fig. 1 A.^6,40,41^ We found that CJ215 underwent a spectral red shifting similar to that seen with ICG when dissolved in fetal bovine serum (FBS), Fig. 1 A.^42^ This red shift in FBS was noted at a pH of 4, 6, 7.4, 8 and 10 with CJ215 being highly stable in FBS over 150 mins at all tested pHs, but degrading rapidly in dextrose at a pH of 4, **Supp. 2.** CJ215 underwent further red shifting when dissolved in human serum albumin (HSA), and FBS at a pH of 7.4, followed by a slow blue shift of ~5 nm in HSA and FBS over the course of 96h, **Supp. 3.** Little to no degradation (loss of absorption) of CJ215 was seen in both HSA and FBS. Although the NIR emission of the dye has been shown by the manufacturer, the SWIR emission and SWIRFI capability of the dye was yet unknown. To assess this, we dissolved CJ215 in dextrose, as well as pure or diluted (in PBS) defibrinated sheep’s blood to simulate human blood, **Supp. 4.** The peak absorption and emission of CJ215 red shifted by ~25nm in the presence of blood versus dextrose (794 to 818 and 813 to 838 nm, respectively) with SWIR emission extending to ~1550 nm, Fig. 1 B and **Supp. 4.** Additionally, the dye underwent an increase in fluorescence intensity when present in serum or blood across this spectrum, further enhancing its suitability for in vivo imaging.^43^ We assessed the ability of SWIRFI to potentially improve tumor delineation through scattering media, by imaging CJ215 through 5 mm of chicken breast, **Supp. 5.** CJ215 was readily detected at 900 (InGaAs sensor response cutoff), 1000, 1100, 1200 and 1300 nm long pass cut offs (achieved via long pass optical filters) with delineation improving with extended (longer) wavelengths. This improvement is attributed to lower autofluorescence, insensitivity to excitation wavelength (808nm) and reduced stray photon scatter via increased water absorption in this realm of the spectrum, **Supp. 5.**^44,45^

### In vitro assessment:

Following characterization of the spectrum of CJ215 we assessed the level of CJ215 uptake *in vitro* in both 4T1 (murine breast cancer) and HT1080 (human fibrosarcoma) cell lines. We first assessed the subcellular localization of the dye via NIR point scanning confocal microscopy. After 3hrs of incubation in 4T1 single cells and 3D spheroid structures, CJ215 was found to be localized to mobile vesicles inside both cells and spheroids, likely taken up via endocytosis Fig. 1 C, D. To further assess the uptake of CJ215, 4T1 cells were incubated with the dye for 3 hrs at either 4°C or 37°C. At 4°C endocytosis, as an energy-consuming process is impaired.^46^ Indeed, CJ215 uptake was reduced by 39.1% in cells at 4°C, indicating that CJ215 is actively transported into cells, Fig. 1 E. We also assessed the effect of inducing apoptosis via staurosporine (Sta) on CJ215 uptake in HT-1080 (fibrosarcoma) cells. The addition of staurosporine (Sta), an apoptosis inducer, increased CJ215 cellular uptake by 99.3% compared with untreated control cells, while addition of z-VAD FMK, an inhibitor of Sta induced apoptosis, reduced that uptake by 48.8% (to 51.1%) compared to Sta alone, Fig. 1 F.^47^ ICG incubation under comparative conditions did not yield any significant change in cellular uptake, Supp. 6. Finally, we found that serum-starved 4T1 cells had a 54.3% higher uptake of CJ215 than cells incubated in the presence of serum, as has been shown for other dyes, **Supp. 7.**^43,48^ Following fixation with PFA, cells retained CJ215 fluorescence levels up to 9 days post fixation, **Supp. 8.**

### In vivo screening:

Having determined that induction of apoptosis increased cellular uptake of CJ215 and that the dye was predominantly taken up by cells via active transport, we next assessed CJ215’s capability for preclinical tumor screening across four tumor lines *in vivo*, in mice implanted with a single lesion site. With lower apoptosis levels found in healthy/normal tissues, we hypothesized that tumors would be the main site of CJ125 retention. We utilized a SWIRFI system equipped with an 808 nm laser (300 mW/cm^2^) to assess CJ215 uptake in male and female mice, across FoxN1^nu^, SCID and BALB/c mouse lines under ambient lighting conditions.^41^ The tested tumor lines comprised xenografted mice bearing breast (4T1, murine, orthotopic), prostate (PC3-PSMA, human, heterotopic), HT1080 (fibrosarcoma, human, orthotopic) and CT26 (colon, murine, heterotopic) tumors, Fig. 1 G. All mice were imaged every 24 hr from 1 to 96 hrs and finally at 144 or 168 hrs post injection, Fig. 1 H. The SWIRFI system (>900nm, sensor response) enabled video rate (30 Hz) acquisitions under ambient lighting conditions and readily achieved sufficient SNR (>5dB) at all tested timepoints with exposure times as low as 1 ms, Fig. 1 H. In all cases the dye showed highly selective tumor uptake, with sufficient SNR achieved within 1 hr post injection. Contrast (CNR), the ability to delineate the tumor from healthy tissue achieved sufficient levels (>3dB, Rose criterion) from 24 to 168 hrs, with CNR increasing in all cases with elongated clearance time, Fig. 1 I.^49,50^ Images from all mice at all timepoints are also presented, **Supp. 9–12.**All SWIRFI presented throughout this manuscript was performed with the ambient lighting LED on, at no detriment to image delity.^41^

### Tumor resection:

**Figure 2:**
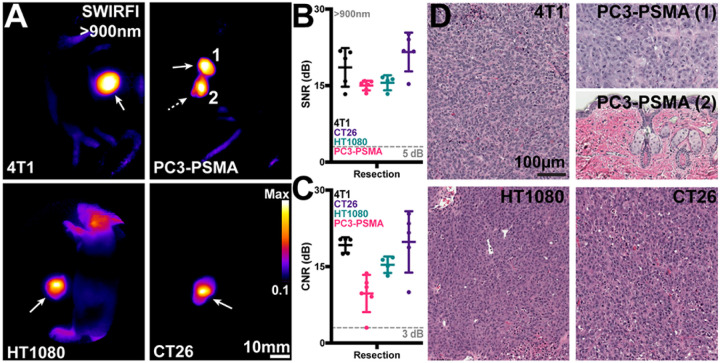
Tumor resection using SWIRFI (>900nm, sensor response) and CJ215. **A)** Resection confirmation of various tumors in euthanized mice. In all cases the primary tumor site is highlighted by the solid arrow, with remnant lesions highlighted by the dotted arrow. In the PC3-PSMA cohort, resection was performed twice on one mouse to completely resect all tumor areas (1, 2). **B)** SNR quantification of all resected lesions, with sufficient contrast achieved in all cases (>5dB threshold). **C)** CNR quantification of all resected lesions, in all cases sufficient CNR was achieved (>3dB threshold) for all primary tumor sites. In all cases the mean, SD, and each replicate (dots, n=4 mice per tumor line, 5 dots include secondary sites) are shown for all tumor lines. Statistical comparisons were not performed as all tumors fulfilled SNR and CNR thresholds. **D)** H&E staining of tumor areas removed during resection, images shown at 20x magnification. All fluorescent areas were confirmed to be tumorous as characterized by the high density of nuclei. For the remnant PC3-PSMA tumor (2) removed during R2, this was determined as residual primary tumor tissue below the skin surface.

post-euthanasia tumor resection was confirmed in all mice using SWIRFI again under ambient lighting conditions. The assumed primary tumor was resected, and both the excised tumor and remaining tissue (i.e., the entire body) were placed side by side within the SWIRFI system, Fig 2 A. In all cases, the primary site contained enough fluorescence to delineate the tumor, while also confirming that the tumor bed was free of residual fluorescence. If suspected remnant tumor was left behind, as noted by highly fluorescent areas this was then resected. For all tested tumors the primary and potential secondary sites had sufficient SNR, Fig 2 B, for detection along with sufficient CNR, Fig. 2 C, to be delineated from the tumor bed site. Resection images for all mice and all tumors are also shown, **Supp. 13–16.**

### Extended spectral emission of CJ215 assessment:

**Figure 3:**
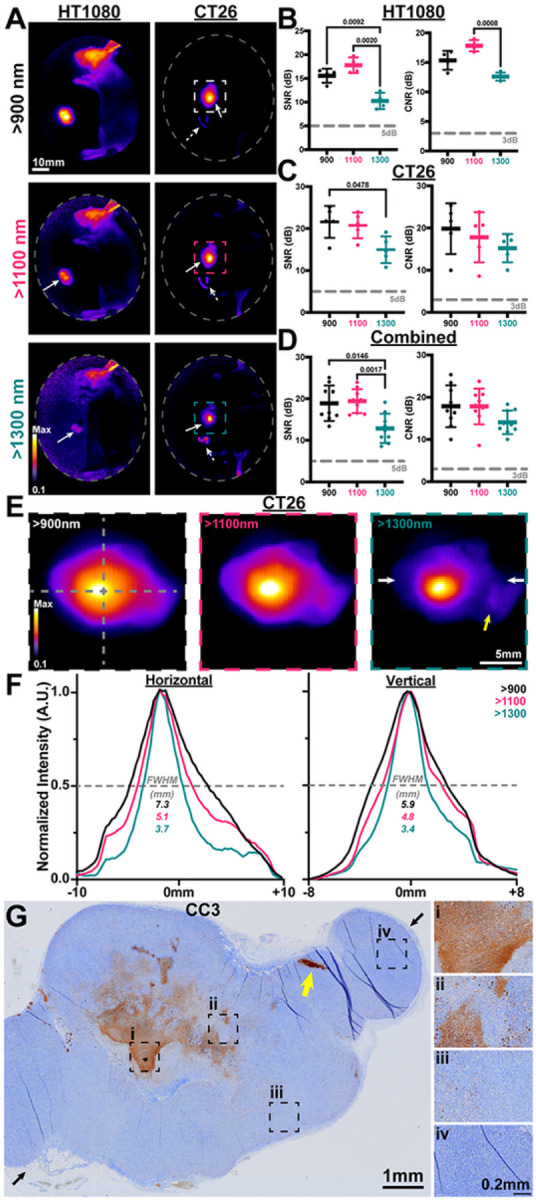
Extended spectral emission (>1100, >1300nm) assessment of CJ215 via SWIRFI. **A)** Extended spectral emission assessment of CJ215 and SWIRFI for resection at >900 (spectral response of the sensor), >1100, and >1300 nm cutoffs (long pass optical filters) for HT1080 and CT26 tumor lines. Note, the core of the tumor becomes most prominent >1300 nm. Non-specific uptake (lymphatic) is seen in the head of the HT1080 mouse. Primary tumor sites are highlighted (solid arrow) along with suspect secondary sites (dotted arrows). **B)** SNR and CNR quantification of the HT1080 tumor line, with >1100 presenting the highest contrast but not significantly improved over >900 nm. **C)** SNR and CNR quantification of the CT26 tumor line, with no difference found in CNR between tested cutoffs. **D)** Combined assessment of HT1080 and CT26 with >1300 nm found to provide the lowest SNR compared to other cutoffs with no significant difference in CNR found when combining both groups. In all cases the mean, SD and replicates are shown (n=4 per tumor line, n=8 for combined) with statistical analysis shown based on a non-paired or matched Welch and Brown-Forsythe ANOVA with Dunnet T3 multiple comparisons test. Thresholds are shown as before (5dB and 3dB for SNR and CNR, respectively). **E)** Zoomed in view of the CT26 tumor at 900, 1100 and 1300 nm. **F)** Vertical and horizontal full width at half maximum (FWHM) assessment (dotted grey lines, E) of the tumor at all wavelengths. The qualitative improvement in tumor core delineation seen at >1100 and best at >1300 nm, is also observed quantitatively. **G)** Corresponding cleaved caspase 3 (CC3) immunohistochemistry staining of the CT26 tumor shown in A and E, showing highest levels of apoptosis at the tumor core with positively stained cells present throughout the sample (i-iv). Black arrows show the approximate slice location with corresponding white arrows in E.

The extended spectral emission of carbocyanine dyes into the SWIR spectrum, which only SWIRFI systems can detect, provides improvements in delineation and resolution, especially through scattering media.^44,51^ We compared the extended spectral emission of CJ215 in HT1080 and CT26 tumor uptake at >900 (sensor response), >1100, and >1300 nm (via long pass optical filters). This was performed to assess increased resolution and penetration via water absorption, which begins to increase >1100 and further increases >1300 nm, Supp. 5.^45^ In all cases CJ215 could be detected but as expected, required increasingly longer exposure times at longer wavelength cutoffs (1–2 ms for >900 nm, 100–200 ms for >1100nm and 500–1500ms for >1300nm cutoffs). Tumor SNR was sufficient at all timepoints, with CNR permitting tumor delineation for HT1080 tumors at 1 hr but not for the CT26 line, Supp. 17. Data combined from both lines, permitted a clearer SNR and CNR assessment and comparison between timepoints. The effect of these cutoffs for resection efficacy was also assessed in both primary sites and potential secondary locations, Fig. 3 A. In all cases, the >1300 nm cutoff provided the lowest SNR and CNR, with >1100 and >900 performing comparably Fig. 3 B,C. a similar trend was identified for screening, Supp. 17. However, the >1300 nm cutoff did improve delineation of the tumor core for both tested tumor lines, Fig. 3 A, E, F. We found dye localization and core delineation improved both qualitatively and quantitatively at extended wavelengths, most obviously at 1300nm, Fig 3 E, F. Using immunohistochemistry (IHC) for cleaved caspase 3 (CC3, a known apoptosis marker) we confirmed that the core of the tumors had the highest levels of apoptosis, with increased apoptosis observed throughout the tumor, Fig 3 G.^52^

### CJ215 biodistribution:

**Figure 4:**
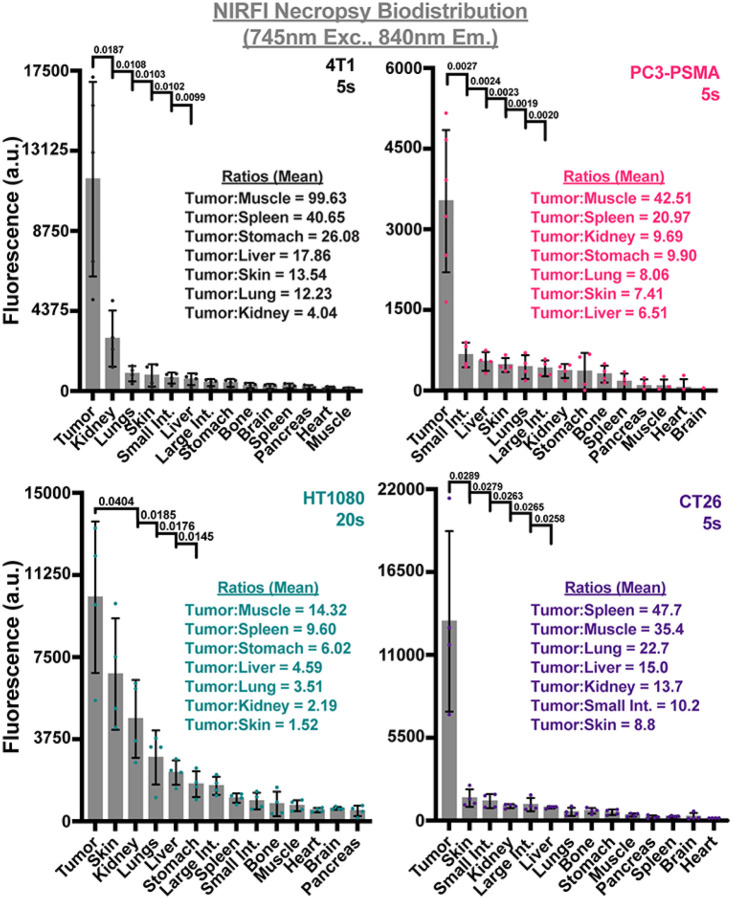
NIRFI (745nm excitation, 840nm emission) necropsy biodistribution assessment of CJ215 in four tumor models. Following resection various organs were harvested for necropsy based biodistribution assessment via NIRFI (IVIS Spectrum, 745nm excitation, 840nm emission) at exposure times from 5–20s. Tumors had the highest level of uptake in all tumor models. Residual kidney fluorescence identifies renal clearance of CJ215. Organs are shown in order of decreasing fluorescence intensity with notable tumor/organ ratios shown. Select p values from unpaired parametric Welch’s t test comparing tumor to organs are shown with the mean (gray bar), SD (black bars) and individual replicates shown (dots, n=4 mice).

**Figure 5:**
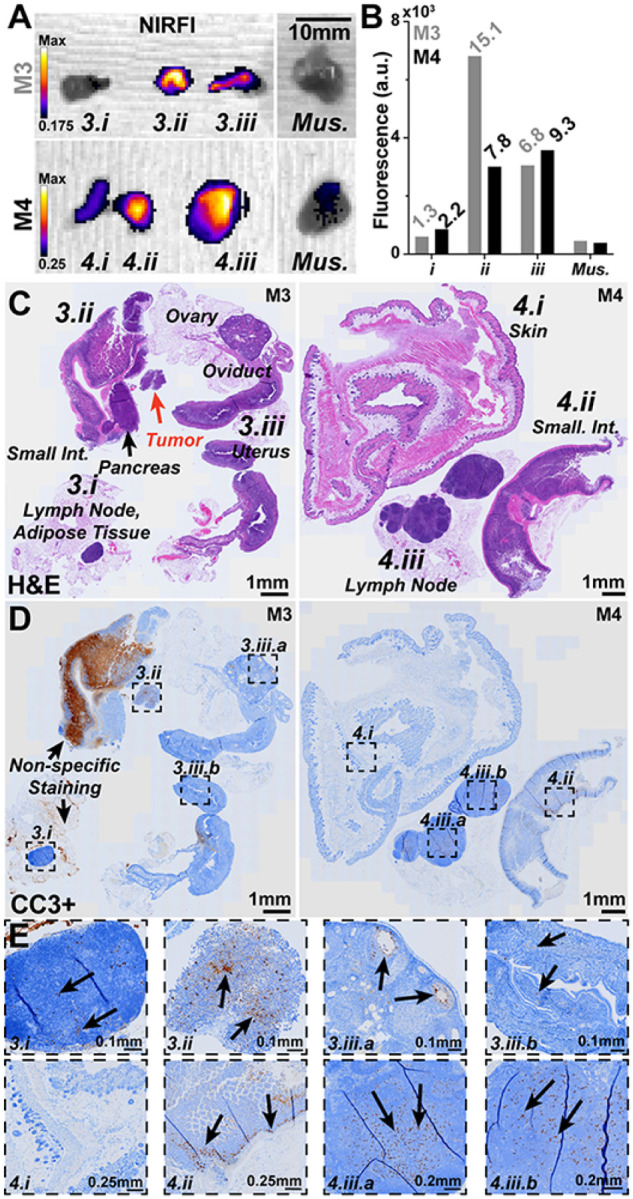
Necropsy and histological analysis of additional regions of interest during CT26 tumor resection. **A)** During CT26 resection and necropsy small areas (labelled as 3.1, 3.2, etc.) were identified and found to be highly fluorescent (NIRFI, IVIS Spectrum, 745nm excitation, 840nm emission) over background areas and muscle tissue. **B)** Quantification of ROIs with ROI to muscle ratios shown (italics). **C)** Left, H&E staining of resected tissues from M3 identified (clockwise) as a lymph node surrounded by adipose tissue (3.1), the small intestine with pancreas and a small neoplastic tumor area not bound to any identifiable tissue (3.2) and finally the reproductive organs including the uterus, oviduct, ovaries (3.3). Right, H&E staining of resected tissues from M4 identified (clockwise) as the skin and subcutis (3.1), a lymph node surrounded by adipose tissue (3.2) and finally the small intestine also with adipose tissue (3.3). **D)** Cleaved caspase 3 positive (CC3+, for apoptosis i.e., damaged cells) IHC staining on a consecutive slice for each mouse sample. Labelled boxes highlight ROIs with increased levels of CC3 specific staining. Non-specific CC3 staining widely seen in the small intestine of M3 should be ignored. **E)** Zoomed in areas as highlighted at various magnification levels: **3.1** Small to moderate number of CC3+ cells were seen in the lymph node cortex and paracortex. **3.2** CC3+ cells were found in the tumor region. **3.3a** CC3+ cells are highlighted in the granulosa of ovarian follicles. **3.3b** Small number of CC3+ cells are seen within the endometrial epithelium. **4.1** No CC3+ cells were seen in the skin. **4.2** Moderate number of CC3+ cells are seen within the crypts with a small number in the enterocytes of villi. **4.3a & 4.3b** Small to moderated number of CC3+ cells were seen throughout lymph node cortex and paracortex.

Whilst SWIRFI is an emerging technique with advantages over established methods, due to the unavailability of imaging systems it remains a restricted resource to many researchers with even fewer clinical options. Additionally, the SWIRFI system employed here had a relatively small FOV, limiting simultaneous imaging of multiple organs. To highlight the suitability of CJ215 for conventional NIRFI, the IVIS Spectrum system (silicon based sensor) was utilized for biodistribution assessment.^53^ Resected organs were imaged simultaneously with fluorescence levels then quantified to highlight the various tumor to organ ratios. In all cases, the tumor was always the brightest tissue, achieving tumor:muscle ratios up to 99.63, Fig. 4. For nearly all models, minimal to no remnant uptake was seen in healthy tissues, resulting in e.g., tumor:spleen ratios of up to 47.7 or tumor:liver of 17.86, Fig. 4. Residual fluorescence seen in the kidneys highlights the renal clearance of CJ215. Biodistribution from all tumors and all mice are shown along with corresponding H&E staining of select organs, **Supp. 18–25.** During the resection of CT26 tumors, additional fluorescence was determined deeper and in proximity to the resected tumor, Fig. 5 A. In two mice (M3, M4) additional areas of increased CJ215 fluorescence were identified below the resected tumor. These areas were also isolated (Fig. 5 B (ROI-2)) and sent with the primary tumor and select organs sent for histological analysis, Fig. 5 C, D, E. When compared with tumor tissue, these tissues had lower CJ215 uptake (but still elevated over other organs) and were found to be tumor free. These areas were identified as small intestine, lymph node, uterine and ovarian tissue with the additional focus in M3 containing a small tumor fragment. Further, IHC staining for cleaved caspase 3 (CC3) Fig. 5 D, an established marker for apoptosis, found increased levels of apoptosis in these tissues. As expected, CT26 primary tumor sites had highly elevated levels of CC3 positive cells, especially within the core of the tumor, **Supp. 26.** The additionally resected tissues which had increased CJ215 fluorescence also displayed increased levels of CC3 positive cells, Fig 5 E.^52^

### Contrast-based SWIRFI for binary tumor delineation:

**Figure 6:**
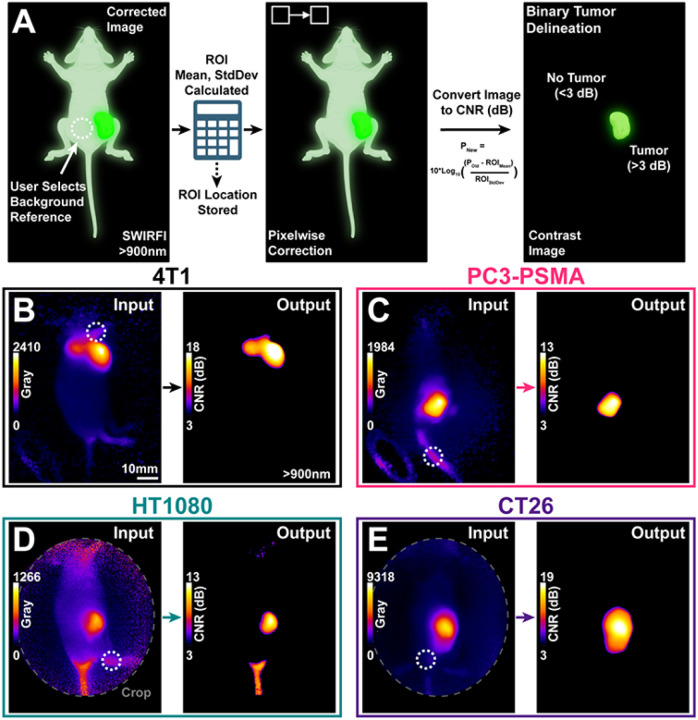
SWIRFI (>900nm) and CJ215 enable contrast-based image generation for binary tumor delineation. **A)** Schematic describing the computation of contrast images, based on user input with >900nm SWIRFI images. Made with Biorender.com. **B-E)** Representative SWIRFI input and output images for all tested tumor lines, >900 nm. The CNR threshold of 3dB was found to be effective for all tumor lines at the 144/168 hr timepoints. The white dotted circle represents the user chosen background reference point. Presented images are single frames from post-processed (corrected) video rate acquisitions, the entire videos are available in Supplemental Video 3. Non-specific uptake is present at the tail of the HT1080 mouse, but the tumor is delineated over surrounding tissue.

Having determined that simply letting CJ215 clear from healthy tissues improved tumor delineation, we utilized further image processing to improve tumor delineation. This was done with the goal of presenting a surgeon with an efficient method of image display when using CJ215, based upon their selection of a non-tumor background reference point. The entire process was completed using ImageJ with the chosen region recorded, the mean and SD of the ROI calculated, followed by respective framewise and pixelwise correction according to widely established CNR calculations, Eq.1. The resulting image is then displayed as a contrast-based image expressed in CNR (dB), dubbed “contrast mode”, Fig 6 A. SWIRFI and CJ215 CNR images can readily provide a surgeon with binary tumor delineation with a 3dB threshold (Rose criterion) found to be effective for all tested tumor lines as seen in Fig 6 B.

1. $${\text{Pixel}}_{\text{New}}=10*{\text{log}}_{10}\left(\frac{{\text{Pixel}}_{\text{Old}}-{\text{ROI}}_{\text{Avg}}}{{\text{ROI}}_{\text{Std}}}\right)$$

### Assessment of CJ215 in a metastatic tumor model:

**Figure 7:**
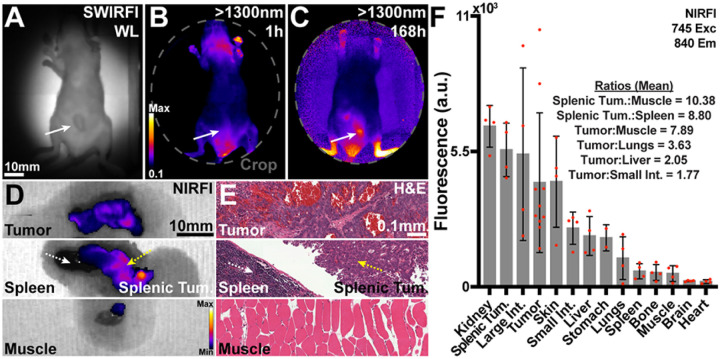
CJ215 assessment in a colorectal peritoneal carcinomatosis model (SW1222). **A)** SWIR reflection image (940nm) of a mouse bearing SW1222 within and protruding through the peritoneum (white solid arrow). **B)** >1300nm image 1h post CJ215 administration. **C)**>1300nm image at 168h post CJ215 administration. **D)** Representative NIRFI (840nm) necropsy image of select organs (M4) including the spleen (dashed white arrow), a tumor mass bound to the spleen/pancreas (dashed yellow arrow) and muscle tissue. **E)** Representative H&E staining of the tissues as shown in B confirming necrotic tumor tissue, spleen, and adjacent tumor (less necrotic) and muscle tissue. **F)** Quantification of dye distribution in the SW1222 model. Select tumor to organ ratios are provided with CJ215 highlighting its renal clearance. Splenic adjacent tumors were quantified separately and compared to both the spleen and muscle tissue. Organs are organized in order of decreasing fluorescence intensity with select ratios, the mean (gray bar), SD (black bars) and individual replicates shown (red dots, n=4) shown.

The four previously four tumor models are readily and widely used for the assessment of tumor targeting of both fluorescent, radioactive, and treatment agents. However, these models represent a single tumor location near the skin surface, where CJ215 could be readily visualized. One significantly more challenging location and urgent clinical problem is the surgical removal of peritoneal carcinomatosis.^54,55^ SW1222 tumors are a human derived colorectal carcinoma tumor line which is highly aggressive and spreads throughout the peritoneal space. This heavily invasive and often palliative surgery requires organ removal during surgery whilst the body cavity is inspected via white light investigation for remnant tumor and would benefit significantly from a suitable targeted dye for FGS. This model of metastatic peritoneal spread consisted of SW1222 luciferase expressing patient-derived colorectal cancer cells injected intraperitoneally.^55^ Once sufficient tumor burden was confirmed using luciferase imaging, animals were administered CJ215 as before. SWIRFI was performed at >900, >1100 and >1300 nm cut offs to assess tumor screening. Tumor visualization of the SW1222 model without prior knowledge of lesion location was initially di cult to distinguish over endogenous colon signal, Fig. 7 A, B, C Supp. 27. As before, necropsy biodistribution was performed with NIRFI at 168 hrs post injection, Fig. 7 D, Supp. 28. We found that tumors were widely dispersed throughout the peritoneum were highly necrotic (essentially dead tissue), with patchy fluorescence seen across identified tumors, Fig. 7 B, C. In all mice, highly fluorescent lesions were seen adjacent to the spleen often engulfing the pancreas (Splenic Tum.), Fig. 7 D and were confirmed to be tumorous via H&E staining, Fig. 7 E, Supp. 29. Highly necrotic tumors still achieved a tumor:muscle ratio of 7.89, Fig. 7 D. Splenic tumors had an improved tumor:muscle ratio of 10.38 and 8.80 over the spleen itself Fig. 7 D, appearing less necrotic (H&E staining, Fig. 7 E), highlighting the ability of CJ215 to delineate organ bound tumor masses and uptake in viable tumors.

### Wound monitoring:

**Figure 8:**
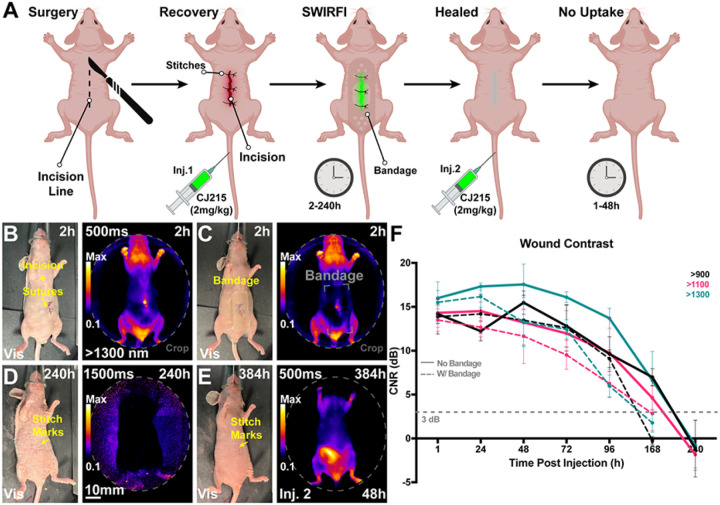
Non-invasive, non-contact and ambient light-resistant wound monitoring through commercially available bandages via SWIRFI (>1300nm) and CJ215. **A)** Schematic representing the experimental protocol including the incision, suturing, injection, and imaging timepoints. Mice received a second injection once the wound had healed and were then imaged every 24 hrs from 1–48 hrs post this injection. Made with Biorender.com. **B)** Representative visible light and SWIRFI (>1300 nm) images without a bandage 2 hrs post-surgery and CJ215 injection. **C)** The same mouse as in A but with a hydrogel bandage place over the wound area. **D)** Representative images at 240h post-surgery and the rst injection of CJ215 highlighting no remnant wound fluorescence (>1300nm). **E)**Representative images at 384h post-surgery and 48h post the second injection of CJ215 highlighting no wound area uptake (wound has completely healed) **F)** Contrast quantification from all mice at all investigated wavelengths (>900, >1100 and >1300 nm cutoffs) with (solid lines) and without bandage placement over wound area (dotted lines). Wound contrast peaks at 48h post-surgery and post injection, decreasing over time as the wound heals. Bandage application prevented wound delineation at 168 hrs. A 3dB CNR threshold (grey dotted line) was utilized as before. In all cases the mean and SD are shown from n=4 mice with mice being measured immediately before and after bandage application. >900nm video rate imaging is shown in Supplemental Video 4 with all Visible, >900, >1100 and >1300nm images of all mice shown in Supplemental Figures#-#.

During hair depilation in the CT26 model slight skin surface damage was inadvertently caused which then proceeded to form scabs and heal over time. We incidentally noticed high levels of CJ215 uptake in these areas, along with tumor uptake, **Supp. 30.** Over the 168hr imaging period, the wound fluorescence decreased with progressing wound healing but remained unchanged in the tumor. Spurred on by this finding, we assessed the ability of SWIRFI to image CJ215 through a variety of commercially available bandages, **Supp. 31.** SWIRFI readily detected CJ215 through all tested bandages, the majority of which were opaque in visible light. Encouraged by this observation, we next implemented a controlled wound monitoring experiment where mice underwent a skin incision with a scalpel, followed by absorbable stitch closing, and were intravenously administered CJ215. Hydrogel burn bandages were placed over the wound during imaging as they provided a non-stick barrier at the wound site, are highly scattering, hindering visible light wound inspection, and provided suitable intensity losses and SNR changes in phantom testing, **Supp. 31.** Mice were imaged at >900, >1100 and >1300 nm with and without a bandage from 2 to 240 hrs (10 days) post-surgery and CJ215 injection, Fig. 8 A, B and **Supp. 32–35.** The bandage was only placed on mice during imaging and was removed before they awoke to prevent discomfort to mice, inadvertent bandage consumption, or bandage degradation. We found that after a single injection of CJ215 the intensity of dye uptake corresponded with wound healing progression, using CNR as a metric for wound assessment, with >1300 nm providing the best wound CNR, along with the wound being readily delineated through the bandage during the main healing phase, Fig. 8 C. The reduction in signal by the bandage prevented reliable wound delineation at 168 hrs and onwards. Wound CNR was detectible from 1–2 hours post injection, highlighting the quick uptake and targeting to wounds of CJ215. CNR peaked 48 hrs post injection then dropped over time until the wound was no longer detectible i.e., completely healed, with longer exposure times utilized between 72 & 240 hrs post injection to ensure su cient signal collection. CNR was found to provide a quantitative metric for wound healing assessment in line with visible light inspection of wound healing but, importantly, also useable when removal of wound dressing is detrimental to the patient. To confirm lack of wound uptake once healed, mice then received a second injection of CJ215 and were imaged every 24 hrs from 1 to 48 hrs post injection. As opposed to right after the initial surgery, sufficient contrast could not be detected within the now healed wound region in all mice, with necropsy based biodistribution showing no significant difference in fluorescence intensity in the wound area versus normal skin, **Supp. 36.** H&E further confirmed the normal physiology of the wound area, **Supp. 37.**

## Discussion

In this work, we have validated the use of the commercial carbocyanine dye, CJ215, for tumor delineation in preclinical cancer models as well as monitoring of naïve and dressed wounds. CJ215 fulfills many requirements for clinical translation and importantly, is optically like ICG and therefore compatible with the wide array of already existing clinical FGS systems, Fig 1 A. CJ215 can take advantage of the reduced optical tissue scatter and absorption in the NIR, which aside from FDA approval, attributes the dominance of ICG. Furthermore, CJ215 has a strong SWIR emission tail, Fig 1 B, which provides advantages over NIR and visible emission ranges.^44^ We set out to elucidate the potential tumor targeting capability of CJ215 by assessing its uptake *in vitro*. CJ215 appeared to be endocytosed and spread across multiple mobile vesicles within 4T1 single cells and spheroids, Fig 1 C, D**, Supp. Videos 1, 2**. CJ215 uptake is increased in cells which are actively metabolic, as shown by the incubation at 4° vs 37°C, Fig 1 E. We show that induction of apoptosis (cell death) with staurosporine doubled cellular uptake of CJ215 in HT1080 cells, while apoptosis inhibition tempered this uptake, indicating that the dye is more readily taken up by apoptotic cells Fig 1 F.^47^ Notably, in the presence of FBS (serum) cellular uptake of CJ215 was reduced by over 50%, **Supp. 7**, with CJ215 found to be stable (still fluorescent) in formaldehyde over the course of days, **Supp. 8.**

CJ215 showed excellent uptake in a variety of tumor models *in vivo*, representing some of the most prevalent cancers including breast (4T1), prostate (PC3-PSMA), fibrosarcoma (HT1080) and colon (CT26), Fig 1 G. High uptake was seen in all models across multiple mouse lines, both in male and female mice, with human and murine derived tumors in various orthotopic and heterotopic locations, Fig 1 G. Our previously established ambient light resistant SWIRFI method and CJ215 could delineate tumors within 1 hr post injection, with highly conclusive delineation at 24 to 168 hrs, Fig. 1 H, I.^41^ Tumor contrast steadily increased over time, in line with previous investigations with other carbocyanine based probes,Fig 1 I.^41^ Tumor resection performed on euthanized mice found that SWIRFI with CJ215 enabled confirmation of tumor resection, identification of remnant tumor, and highlighted other suspect regions with the tumor site always having the brightest fluorescence, Fig 2.

SWIRFI enabled investigation of the extended spectral emission of CJ215 which current silicon based sensors cannot detect, **Supp 5.**^6,40,51^ We assessed the reduction in photon scatter of CJ215 by increased water absorption at >1100 and >1300 nm long pass cutoffs to improve tumor CNR, Fig 3. For both screening and resection these wavelengths ultimately did not provide a major advantage in tumor delineation, due to the high tumor uptake of CJ215 and at the cost of non-video rate imaging, Fig 3 B–D. However, the >1300nm cutoff provided both qualitative and quantitative non-destructive insight to CJ215 distribution within the tumor core, highlighting tumor heterogeneity, Fig 3 E, F. This core uptake was reflected in CC3 (apoptosis) staining via IHC, confirming the observations made via SWIRFI, Fig 3 E, G.

SWIRFI has optical benefits but remains a novel and often inaccessible tool due to limited access to suitable imaging equipment. The utilized SWIRFI system has a reduced FOV (a single mouse), making multiple organ comparisons cumbersome. We performed NIRFI necropsy biodistribution post resection on an IVIS system with a larger FOV (up to 5 mice), further confirming both CJ215 tumor localization and suitability for NIRFI, Fig 4. Tumors had the brightest fluorescence in all experiments with non-tumor bearing organs having minimal residual dye uptake. This biodistribution enabled un precedented tumor:muscle ratios of up to 100 where anything above 2 or even 1.5 is considered successful, Fig 4.^56^ Furthermore, CJ215 cleared from vital organs such as the liver, spleen and pancreas enabling tumor:organ ratios in the mid 40s, Fig 4. Although not present in these single site models, this highlights that CJ215 has the potential to delineate various organ metastases with high contrast for improved delineation. Importantly, these ratios were achieved by simply allowing CJ215 to clear from healthy tissue after a single systemic intravenous injection. CJ215 cleared renally as highlighted by the retained kidney fluorescence, in stark comparison to other dyes or nanoparticle-based approaches where liver clearance can be a predominant factor, Fig 4.^15,57–59^ Select organs from these mice were assessed via histopathology (H&E staining), confirming tumor tissue from healthy organs, and assessing additionally resected regions. All suspected tumor regions were confirmed to be tumorous via H&E staining.

During CT26 resection and necropsy, additional areas aside from the primary tumor demonstrated elevated organ:muscle ratios, Fig 5 A, B. These regions were confirmed to be lymph node, uterine, ovarian, colon as well as a single tumor site, Fig 5 C. IHC for CC3 (apoptosis marker) was performed on these tissues, highlighting their increased apoptotic levels, Fig 5 D. This also confirmed the highly apoptotic core seen at the center of tumors, where CJ215 uptake had been brightest, Fig 3 G**, Supp. Fig 26.** The exact cause of cellular damage and apoptosis in these regions is unclear, however they were predominantly near the primary tumor site and may have been compressed as it proliferated, causing cell stress and apoptosis.

Confirmation of the high tumor uptake, seen during SWIRFI screening, spurred us to present images in a way that would be most useful for FGS. Image presentation during surgery is an integral component of FGS when assessing novel systems and probes, and ultimately is where impact and utility of the approach are determined.^60^ Sufficient SNR was always achieved with SWIRFI, the main tumor delineation factor was concluded to be contrast (CNR), as expected, Fig 2 H, I. Here, we present video rate images in a contrast mode where the user selects a non-tumorous background reference point, Fig 6 A. A custom script in ImageJ facilitated this, across SWIRFI videos at >900 nm and achieved pixelwise image correction at a rate of ~40 ms per image, Fig 6 B**, Supp. Video 3**. The 3dB threshold was found to be successful in highlighting tumor areas for all lines, ful lling the Rose criterion.^49,50^ Additionally, the conversion of images from a linear to a logarithmic scale counteracted the high tumor core uptake of CJ215 easing entire tumor delineation, Fig 6 B **CT26 model.** This method enables binary tumor delineation, on the fly for FGS and can be readily implemented by other researchers and for other probes. Graphics processing unit (GPU) implementation (outside the scope of this work, but possible with ImageJ) could decrease image processing time to enable real-time visualization (assuming image handoff from memory to GPU is efficient) during FGS as opposed to in post processing with few ms acquisition times.^61^

With the success and high tumor localization of CJ215 in single site tumor models, we then assessed its potential in a more challenging way. The SW1222 cell line is a human derived, highly aggressive, and metastatic tumor model which spreads throughout the peritoneal space. Current clinical treatment includes surgical removal, where white light visual inspection is utilized in a highly invasive surgery. This procedure comprises organ repositioning with peritoneal inspection and often fails to detect and remove all lesions. CJ215 was assessed in one such model where mice received SW1222 cells injected intraperitoneally, Fig 7 A.^55^ Tumor growth was confirmed with luciferase, but SWIRFI and CJ215 (even at extended wavelengths e.g., >1300nm) did not conclusively delineate tumors through the peritoneum, Fig 7 A**, Supp. Fig 22.** At 168 hrs post injection, SWIRFI resection was attempted with tumors being identified as solid, black necrotic tissue (likely dead), widely dispersed throughout the peritoneum, and bound to the intestines and spleen, Fig 7 B. NIRFI confirmed lower tumor uptake in the most necrotic tissues but did highlight large lesions spread around the spleen and pancreas, which could be readily delineated, Fig 7 B**, Supp. Fig 23**. Even though less successful than single site models, CJ215 achieved tumor:muscle ratios of up to 10 within these tumors with an uneven distribution across highly necrotic areas, Fig 7 C. Due to the rapid growth of these tumors and their widespread dispersion, they lacked viable vascular networks within necrotic tissue at the injection timepoint. The non-existent metabolic activity, and poor blood delivery in dead and pervasively necrotic tissue hinders CJ215 delivery and therefore uptake in such lesions, Fig 7 B**, Supp. Fig 24.**^62^ This is further highlighted by the high presence of disrupted blood vessels and necrotic tissue morphology in H&E staining of main tumors, versus the more viable and less necrotic splenic/pancreatic tumors which presented higher tumor:muscle ratios.

Hair depilation during CT26 tumor imaging inflicted minor wounds. These wounds showed high CJ215 uptake, with CJ215 clearing from them over time as they healed but remaining in the adjacent tumor, **Supp. Fig 30**. This feature of CJ215 uptake is reminiscent of previous descriptions of tumors being wounds which do not heal.^63^ SWIR imaging is commonly used in industrial settings to detect e.g., liquids through substances which are opaque in the visible spectrum and therefore also opaque to the human eye.^39^ We combined this aspect of SWIR sensors with SWIRFI to detect CJ215 through a variety of commercial bandages in phantoms, **Supp. Fig 31.** Hydrogel bandages, used in burn wound healing, were chosen to track wound healing in mice post a controlled incision, Fig 8 A, B**, Supp. Video 4.**^64,65^ Similar to tumor delineation, CNR could be inversely used to elucidate wound healing progression completely noninvasively after a single systemic injection of CJ215, Fig 8 C. CNR increased up to 48hrs post injection and then decreased as wounds healed in line with visible inspection (without bandages) with the >1300nm cutoff providing the highest CNR, albeit not video rate, Fig 8 C**, Supp. Fig 32–35.** Having completely healed and following a second injection of CJ215, the wound area showed only minimal uptake, found to be in line with non-injured skin, **Supp. Fig 36.** These mice also provided further biodistribution data of CJ215 in non-tumor bearing conditions, confirming the renal clearance as seen at later timepoints and again the lack of uptake in other organs, **Supp. Fig 37.** This aspect of CJ215 and SWIRFI provide an objective, rapid (seconds), and quantitative solution in chronic wound monitoring with potential clinical applications in e.g., diabetic and burn wound victims.^66^ This will be especially useful where bandage removal may cause pain to victims or pose a risk of infection.^64^ Based on these experiments a patient (even those not undergoing tumor resection) could receive a single injection of CJ215 upon surgery completion with wound healing tracked for days during post-operative care. SWIRFI and CJ215 can readily visualize bandage covered wounds under ambient lighting conditions providing an additional application of clinical uorescence imaging, not limited to only tumor resection.

Throughout this work we have shown that CJ215 is highly tumor selective with excellent clearance from healthy organs. This clearance further aids tumor delineation and even enabled the detection of tumors on or close to non-affected organs. CJ215 displayed notable spectral changes when dissolved in FBS, HSA or blood compared to dextrose eluding to that *in vivo*, CJ215 is binding to serum and other components of blood. This is evidenced by the shift in absorption peak of CJ215 in dextrose, HSA, FBS and blood (795, 805, 810 and 818nm, respectively **Supp. Fig 2–4**), with each solution presenting an increasingly convoluted environment and more components for CJ215 to bind to, more akin to *in vivo* settings. Importantly following an initial red spectral shift, CJ215 underwent a blue shift over the course of days (~5–10nm) potentially forming a covalent adduct with albumin and blood components, as shown for other carbocyanine dyes.^43,48^ In comparison to previous work, CJ215 contains a methoxy group on the cyclohexenyl ring at the center of the heptamethine bridge as opposed to a chloride group along with some other subtle chemical differences.^43^ This methoxy group and likely CJ215 as a whole is less reactive than a carbocyanine dye with a chloride group at the same location, explaining the slow blue shift (days) in the presence of blood components. Based on the biodistribution with renal clearance and *in vitro* experimentation, CJ215 is likely binding to blood components *in vivo* which are then preferentially taken up by apoptotic and stressed cells including tumors and wound healing areas.^48^ This is further confirmed by the serum free *in vitro* experiments and biodistribution similarity to radiolabeled albumin, with protein catabolism by tumors and wound healing being a predominant driver of uptake.^43,67^

One limitation of CJ125 is that based on our experimentation, CJ215 will likely not be of use in highly advanced and necrotic (dead) tumor tissue with poor vasculature as shown in the SW1222 model. However, surgery would not be performed at this level of tumor burden. This is unlike the tested single site tumor models, where CJ215 is readily delivered via the blood stream and vasculature, dispersed throughout the tumor itself and then trapped within highly apoptotic cores. Nevertheless, the SW1222 model did validate the ability of CJ215 to delineate peritoneal metastasis in multiple dispersed lesions. Due to the ability of CJ215 to highlight and be retained in apoptotic tissue, CJ215 may not be suitable for FGS in patients which have already received e.g., adjuvant chemotherapeutics which are known to cause off target cell death.^68^ This would likely increase non-tumorous background uptake, potentially reducing tumor CNR or leading to false positives as seen in the CT26 model, however, uptake in the tumor could be increased as well. Conversely, due to the low uptake in healthy tissue and established biodistribution seen here, CJ215 could be used to study these affects and the organ protection provided by co-administered compounds. Fortunately, surgery is often the first treatment for most solid tumors, highlighting the suitability of CJ215 for tumor FGS within current surgical guidelines. Whilst toxicology has not yet been confirmed, no adverse events were reported in any of the mice used herein with all mice surviving the injections and main organs appearing histologically normal. Repeated injections also caused no issue and as CJ215 is renally cleared, it may reduce potential liver toxicity.

In summary, CJ215 provides a unique dye for tumor and wound monitoring through binding to mostly apoptotic cells, enabling delineation of various tumors in murine models (all tested models have been included and we have yet to find a solid model which CJ215 does not delineate) and quantitative wound monitoring. These aspects position CJ215 as a pan cancer agent, with high tumor selectivity for multiple applications reducing the often-limiting factor of FDA approval pipeline costs and bridging the “valley” between preclinical and clinical translation. Finally, CJ215 is a commercial product facilitating rapid replication of these results and further investigation by any researcher with a suitable device e.g., IVIS. This is in stark comparison to the burden shouldered by academic labs where mass production of custom compounds is often unachievable and not a focus.

## Materials And Methods

### Spectral Analysis:

CJ215 (Proimaging, Paris, France) absorption spectra were determined after dissolving in either dextrose (5% Dextrose Inj. USP, 100/150 mL PAB^®^, NDC 0264–1510–32, Braun Medical, USA), fetal bovine serum (FBS) at varying dye concentrations between 0.2 and 20 μM was assessed using an ultraviolet-visible/NIR spectrometer (Jasco V-780, Jasco Inc., USA) 100 μL cuvettes using a dye free vehicle sample for correction, with readings displayed in optical density (OD). Extended SWIR tail emission was recorded using the same solutions on a 96 well optical plate (UV Half Area Plate, 3679, Corning, USA) with a custom SWIR fluorescence spectrometer, as previously described.^41^ Briefly an 808nm laser excitation source, inverted microscope (IX-71 microscope, Olympus, USA) and 20x SWIR lens (Olympus, USA) were used to collect spectra, with intensity artefacts corrected appropriately. Dextrose, FBS, HSA (human serum albumin, H6914–20ML, MilliporeSigma, USA) or defibrinated sheep’s blood (R54004, ThermoFisher Scientific, USA) were used to assess the spectral absorption and emission shift of CJ215 in solution over time, measured on a microplate (Corning^®^ UV-Transparent Microplates, CLS3679, MilliporeSigma, USA) on an automated plate reader (SpectraMax ID5, Molecular Devices, USA). Solutions were incubated in either 1.5 ml Eppendorfs or 15 ml tubes at 37°C at 300 RPM (Thermomixer R, Eppendorf, USA).

### In vitro assessment:

4T1 (ATCC^®^ CRL-2539) cells were cultured in RPMI media (supplemented with Pen-Strep and FBS) until confluent in 6 well plastic poly-d-lysine coated plates with an embedded glass cover slip (P06G-1.5–10-F, MatTek, USA). In these conditions 4T1 cells formed a mix of single cells and spheroid aggregations. Cells were washed once with fresh medium, then incubated (incubator, 37°C, 5% CO_2_) with CJ215 (suspended in cell culture medium) for 3 hrs at a concentration of 0.645 mM. 1 μl of stock DAPI solution (62248, Thermo Fisher Scientific, USA) was added to each well at 2hrs 40 mins into CJ215 incubation. At 3hrs well media was aspirated, cells washed once with fresh medium and then 1 ml of medium was added to each well. Point scanning confocal microscopy was then performed (STELLARIS 8, Leica, USA) within an hour, recording various images (stacks, videos) with appropriate DAPI, CJ215 and polarized white light settings. Images were imported into ImageJ, split by color with gaussian blur (sigma = 1) applied to DAPI and CJ215 channels followed by contrast adjustment for easier visualization prior to remerging the channels.

Active transport was assessed also using 4T1 cells incubated in pre-coated plastic 6 well plates (no glass coverslip). Cells were washed once and then placed into either a fridge or incubator for 10 mins prior to incubation with CJ215 as performed for confocal imaging. CJ215 uptake was assessed using the 700 and 800 channels on an Odyssey CLx, with image analysis performed on .tiff images consisting of determining the mean fluorescence within each well (Fiji/ImageJ).^69^ HT1080 (ATCC^®^ CCL-121^™^) cells (75,000 cells/well) were seeded on a 24 well plate with 500 μL of cell medium (DMEM, with Pen-Strep and 10% FBS). After 24 hours, the cells had attached to the plate surface and Staurosporine was added to the wells with a final concentration of 2 μM Staurosporine. The cells were incubated with Staurosporine for 18 hours. 5 μL CJ215 stock solution (0.5 mg/mL) was mixed with 1.2 mL media and 50 μL dye-media mix was added to each well. Dye was incubated with the cells for an additional 6 hours, then cells were washed with PBS once. The plate was imaged and quantified as before (Odyssey CLx and Fiji/ImageJ). Serum free and serum uptake of CJ215 was assessed with 4T1 cells plated on 6 well plates, as before. Cells were starved in serum free conditions (cell medium without FBS) for 3 hours prior to addition of and incubation with CJ215, also as before. Cells were washed once with respective media and then fixed and imaged in 1 ml of formaldehyde solution (formaldehyde solution 4%, microscopy grade, 1.00496.5000, Sigma-Aldrich, USA) and imaged as before (Odyssey CLx). Formaldehyde stability of CJ215 was assessed with the same cells stored at 4°C for up to 9 days post fixation, again imaged as before.

### In vivo experimentation:

Single tumor model sites were generated with multiple cells, mouse lines and sexes. In brief, 3.0×10^5^ 4T1 cells suspended in 30μL of Matrigel were injected into the mammary pad of female FoxN1^nu^ mice. 5×10^6^ PC3 cells expressing human prostate specific membrane antigen (PC3-PSMA) suspended in 50μL of PBS were subcutaneously injected into the flank of male NOD.Cg-Prkdcscid/J mice. 5×10^6^ HT1080 cells suspended in 100μL of 50:50 Matrigel and PBS were subcutaneously injected into the ank, at the top of the leg in female FoxN1^nu^ mice. 2×10^6^ CT26 cells suspended in PBS were subcutaneously injected into the flank of female BALB/cAnN mice. Mice were utilized at various ages, with tumors allowed to proliferate until tumors at various sizes could be visually determined, approx. >50 mm^3^. 5.0×10^6^SW1222 cells expressing luciferase suspended in 100 μl of cell medium were intraperitoneally injected into female FoxN1^nu^ mice, sporadically forming tumor lesions, and assessed using luciferin. Female FoxN1^nu^ mice (bearing no tumors) were also used for wound healing experiments.

In all cases mice received a 2mg/kg intravenous injection of CJ215 suspended in clinical grade dextrose. Upon receipt of CJ215 it was suspended in dextrose, aliquoted into glass dram vials and lyophilized for storage at −20°C. Injection solutions were prepared within 1 hr prior to administration, with solutions appearing dark green. The dye did not form any noticeable clumps, could be readily resuspended in dextrose simply using a pipette and animal injections went smoothly. CJ215 was not handled in any overly restrictive or particular manner and no adverse event was noticed post dye injection. All mouse handling, experimentation, imaging, and housing was performed according to NIH guidelines and via approved IACUC protocols at MSKCC. Mice received food and water *ad libitum*, under a 12 hr on/off light cycle, 5 mice per cage with food. FoxN1^nu^ mice used in tumor experiments were fed either a solid amoxicillin or Sulfatrim based diet to counteract skin infections. SW1222 and wound healing mice were placed on a low autofluorescence diet to counteract observed intestinal signals, with wound healing mice receiving Sulfatrim water to counteract skin infections. Surgery was performed on mice in line with preapproved protocols with mice having anesthesia induced at 3% v/v followed by maintenance at 1–2% v/v in oxygen and placement on a heating pad. Prior to surgery the mice received subcutaneously received buprenorphine (0.5 mg/kg) and meloxicam (2mg/kg) with bupivacaine (0.1cc) administered at the incision site for numbing. The surgical eld was cleaned for surgery using sets of alternating scrubs, (alternating either povidine-iodine (Betadine), chlorhexidine and 70% isopropyl alcohol). All surgical instruments were sterilized by steam sterilization and using a glass bead sterilizer between animals. A scalpel was used to create a ~1cm incision with wound sealing performed with mono lament absorbable suture (monocryl). Mice were monitored post recovery every 24hrs and received meloxicam (2mg/kg) subcutaneously for up to 72hrs post-surgery.

Tumor resection was only performed on mice which had been euthanized via CO_2_ inhalation, in line with approved protocols. Following this the tumor was resected under white light visualization with resection confirmed using SWIRFI. Necropsy biodistribution was assessed by isolating the following organs: tumor or wound site, liver, kidneys, lungs, brain, spleen, pancreas, stomach, small and large intestines, skin, bone, muscle, and heart.

### SWIRFI and NIRFI:

Ambient light resistant SWIRFI was performed as previously described.^41^ In brief, a hyperspectral SWIR system (IR-VIVO, Photon etc., Canada) equipped with an 808 nm laser (~300 mW/cm^2^) for dye excitation, a 940 nm LED for SWIR white light (reflectance imaging) and an RGB LED was used for SWIRFI. The ambient lighting LED was on for all SWIRFI images presented throughout this manuscript. Extended emission imaging was achieved using suitable long pass optical filters (Thorlabs, NJ, USA) mounted in front of the lens of the system in a custom mount. Exposure times ranged from 1–1500 ms with data stored in a .h5 format. Images were converted to tiff les with corrections then performed in Fiji/ImageJ as before including dark noise subtraction, outlier removal, and cropping.^41^ Defined ROIs were used to assess brightness, SNR and CNR, also as previously described.^41^ NIRFI for biodistribution was performed using an IVIS Spectrum (PerkinElmer, USA) with ICG based lter sets (745 excitation and 840 nm emission), at exposure times of 5–20s, high lamp, f# of 1 and small binning. Organ quantification was performed in ImageJ/Fiji with ROIs drawn across each organ in the raw luminescent tiff le exported from the IVIS. This system was also used for luciferase imaging of SW1222 mice in bioluminescence mode. For all live animal imaging, mice were anaesthetized using isoflurane inhalation at an induction of 3% v/v and maintenance of 1–2% v/v in O_2_ and were placed on a heating pad during imaging.

### Histological Assessment:

Paraffin embedded organs (tumor or wound, liver, kidney, spleen, and muscle) were prepared as previously described to generate either on 5 μm thick unstained or H&E stained (Leica Autostainer, ST5010) slides.^41^ H&E slides were imaged using an automated slide scanner at 20x magnification (0.8 NA, 3DHistech, Budapest, Hungary) with representative images shown at 20x (SlideViewer, Version 2.5, 3DHistech). Immunohistochemistry for CC3 was performed on paraffin sections using a Leica Bond RX automated stainer. After heat-induced epitope retrieval in a pH 6.0 buffer, anti-CC3 antibody (Cell Signaling 9661) was applied at a concentration of 1:250, followed by a polymer detection reagent kit according to the manufacturer’s instructions (DS9800, Novocastra Bond Polymer Refine Detection, Leica Biosystems). The chromogen indicating positive immunoreactivity was 3,3 diaminobenzidine tetrachloride (DAB), and sections were counterstained with hematoxylin. CC3 IHC images were acquired an Olympus VS200 slides scanner and VS200 ASW 3.4.1 software (Evident Scientific, Hamburg, Germany) using a 20X/0.80NA objective to generate whole slide images with a pixel size of 0.2738 μm. CC3 images are presented at various magnifications after visualization in QuPath (v0.4.4) and image export to Fiji/ImageJ2 (version 2.14.0/1.54g).^69,70^ Select H&E and CC3 whole slide images were examined by a board-certified veterinary pathologist (SM).

### Data Analysis:

Image processing and quantification was performed in ImageJ/Fiji with results saved in Excel les (Excel for Mac, ver. 16.57, Microsoft, USA). SNR and CNR values were calculated in Excel with graphing with various statistical analyses as listed performed in GraphPad Prism (Prism 10 for MacOS, GraphPad Software LLC). A custom script (macro) was developed in ImageJ/Fiji for pixelwise correction of SWIRFI images based on a user defined ROI. The script calculated the mean and SD of this ROI for all frames in a video image and uses the CNR formula commonly utilized to present images in “contrast mode”.

### Wound healing assessment:

Assessed bandages in phantom imaging included hydrocolloid gel (Item no. 990443, 33×53 mm, Walgreens, USA), hydrogel (Item no. 973181, 44.5×73 mm or 25×56 mm, Walgreens, USA), transparent (60×70 mm, TEGADERM, Nexcare, 3M, Item no. 666368, Walgreens, USA) and liquid spray (on saran wrap, Item no. 202297, Walgreens, USA). Hydrogel bandages were applied to mice only during imaging, with each mouse receiving a new bandage and the bandage being removed prior to removing the mouse from the imaging setup.

## Data Availability

All data and code required used throughout this manuscript is available from the corresponding authors upon request. Note, all code to process images was peformed in ImageJ via already available plug-ins.
